# Serum neurofilament light chain and multiple sclerosis prognosis: a systematic review and meta-analysis

**DOI:** 10.3389/fimmu.2026.1818869

**Published:** 2026-04-28

**Authors:** Yejun Chen, Minyan Cai, Xudong Lv

**Affiliations:** Department of General Practice, Zhuji People’s Hospital, Shaoxing University, Zhuji, China

**Keywords:** biomarker, meta-analysis, multiple sclerosis, neurofilament light chain, systematic review

## Abstract

**Background:**

Serum neurofilament light chain (sNfL) has emerged as a promising biomarker of neuroaxonal injury in multiple sclerosis (MS), but its prognostic value for clinical and radiological outcomes requires comprehensive synthesis of available evidence.

**Methods:**

In this meta-analysis, we systematically searched PubMed, Embase, Ovid, and Cochrane databases from January 2015 to January 2026 for studies evaluating the association between baseline sNfL levels (high vs low) and MS progression outcomes. Primary outcomes were new clinical relapse (NCR) and ≥1 gadolinium-enhancing (Gd+) lesion. Secondary outcomes included relapse within 1 year (R1Y), new T2 lesions (NTL), and confirmed disease worsening. Random-effects models were used to pool risk ratios (RRs) with 95% CIs.

**Results:**

Among 3,281 identified records, 13 studies (n=12,513 patients) were included. Elevated sNfL was significantly associated with increased risk of NCR (6 studies; RR 1.42, 95% CI 1.30-1.54; P<0.01) and ≥1 Gd+ lesion (5 studies; RR 1.47, 1.39-1.56; P<0.01), with low heterogeneity (I²≤34.9%). Consistent associations were also observed for NTL (4 studies; RR 2.11, 95% CI 1.59–2.64; P<0.01) and disease worsening (5 studies; RR 2.10, 95% CI 1.57–2.63; P<0.01), although the latter showed moderate heterogeneity (I²=60.7%). An association was observed for R1Y (2 studies; RR 1.94, 95% CI 1.11–2.76; P<0.01), although the evidence for this outcome remains limited. No substantial publication bias was detected for the main outcomes, and leave-one-out sensitivity analyses confirmed the robustness of the results.

**Conclusion:**

Elevated sNfL concentrations are consistently associated with increased risks of clinical relapse, radiological activity, and disability progression in MS, supporting its utility as a prognostic biomarker for stratifying disease course and informing clinical trial design.

## 1 Introduction

Multiple sclerosis (MS) is a chronic inflammatory and neurodegenerative disorder of the central nervous system, representing a leading cause of non-traumatic neurological disability in young adults globally (1, 2). The clinical course of MS is remarkably heterogeneous, ranging from relatively benign forms to rapidly progressive disability (3). This profound variability poses a significant challenge for clinicians in prognostication, treatment decision-making, and counseling of patients. While clinical parameters, magnetic resonance imaging (MRI), and cerebrospinal fluid (CSF) analysis provides valuable diagnostic and monitoring insights, there remains an unmet need for accessible, reliable, and dynamic biomarkers that can objectively reflect underlying neuroaxonal injury (4, 5).

Neurofilament light chain (NfL), a key structural component of neuronal cytoskeletons, is released into the extracellular space upon axonal damage (6). Historically measured in CSF, NfL has emerged as a sensitive biomarker of neuroaxonal injury across multiple neurological conditions (7). The recent development of ultra-sensitive single-molecule array technology has enabled the robust quantification of NfL in peripheral blood, transforming it into a minimally invasive and repeatable tool (8). Serum NfL (sNfL) levels correlate strongly with CSF levels and have been shown to reflect disease activity, treatment response, and neurodegeneration in MS (9, 10).

Numerous individual observational studies and clinical trials have investigated the prognostic potential of sNfL, suggesting associations with future relapse risk, MRI lesion activity, brain volume loss, and disability progression (11–15). However, the magnitude and consistency of these associations across diverse MS phenotypes and clinical contexts remain inadequately synthesized (16). A systematic and quantitative synthesis of the available evidence is crucial to establish the precise prognostic value of sNfL, define its role in clinical practice and trial design, and identify gaps for future research. This meta-analysis aims to collate robust evidence to determine the strength of association between baseline or longitudinally measured sNfL levels and key clinical and radiological outcomes in people with MS, thereby evaluating its utility as a prognostic biomarker for disease course and long-term disability.

## 2 Methods

This systematic review and meta-analysis was reported according to the Preferred Reporting Items for Systematic Reviews and Meta-Analyses (PRISMA) guidelines (17).

### 2.1 Study selection strategy

Two authors performed the publications search independently, using PubMed, Embase, Ovid and Cochrane databases. The search was performed to identify all studies comparing the outcomes of MS patients with high sNfL or low sNfL expression who have been diagnosed with MS. The search strategy was based on the following index: ‘Sclerosis, Multiple’, ‘MS (Multiple Sclerosis)’, ‘Sclerosis, Disseminated’, ‘Disseminated Sclerosis’, ‘Multiple Sclerosis, Acute Fulminating’, ‘light neurofilament protein’, ‘neurofilament protein light’, ‘neurofilament light polypeptide’, ‘NEFL protein’, ‘NEFL polypeptide’’, ‘NF-L protein’, ‘NF-L polypeptide’, ‘NEFL protein, human’, ‘neurofilament, light polypeptide 68kDa protein, human’, ‘CMT1F protein, human’, ‘CMT2E protein, human’, ‘NF68 protein, human’, ‘Neurofilament triplet L protein, human’. Only studies on humans were considered for inclusion. There was no language limitation in the studies searched. Reference lists of all retrieved articles were manually searched for additional studies.

### 2.2 Inclusion & exclusion criteria

**Figure 1:**
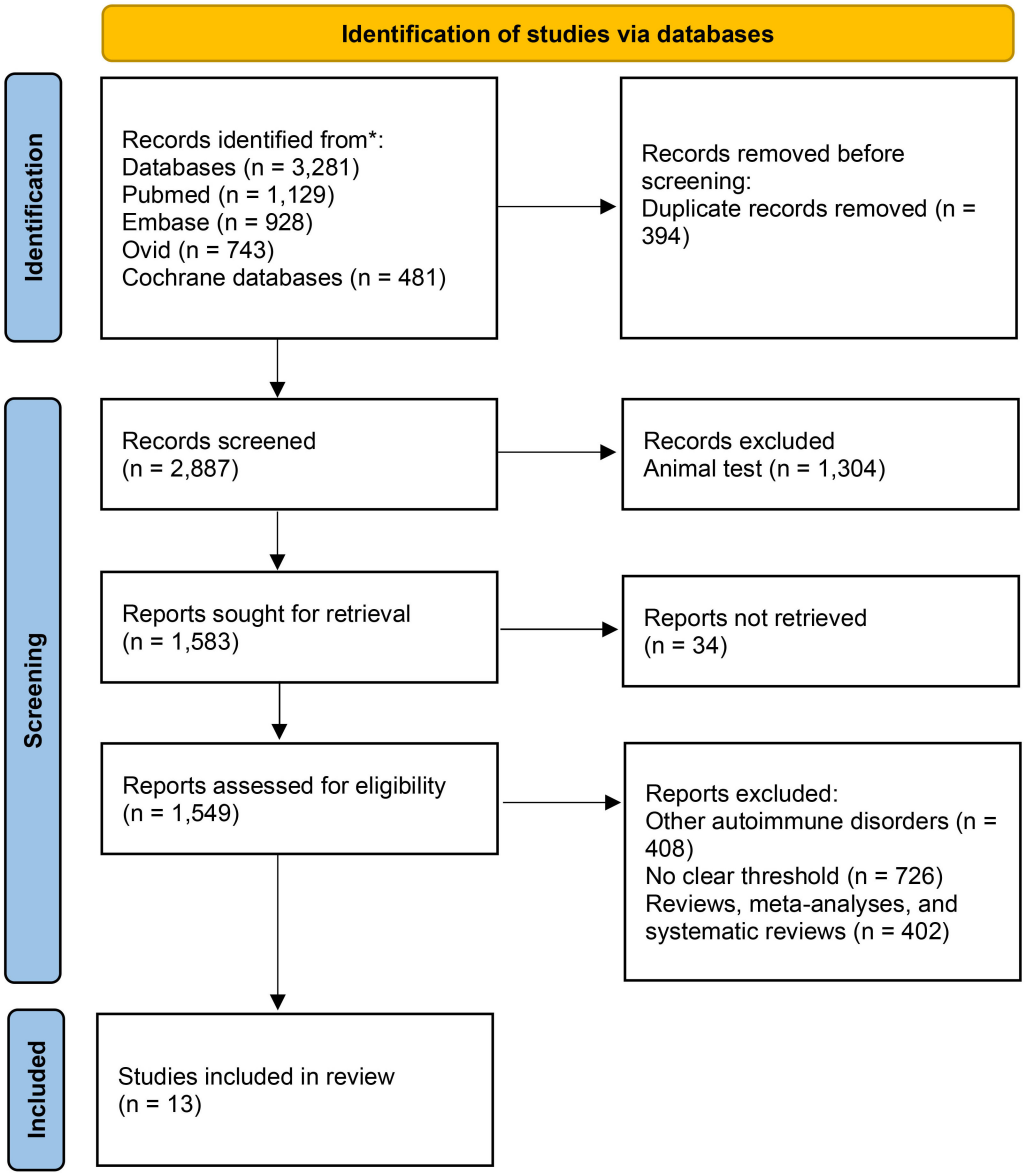
PRISMA flow diagram of the study selection process.

The principles of participants, intervention, control, outcome and study design (PICOS) were strictly adhered to for inclusion and exclusion of studies. This study included studies that evaluated the association between sNfL levels and the prognosis of MS. MS diagnosis in the included studies was based on the revised McDonald criteria, with the specific versions confirmed for each study and corresponding to the time period in which the study was conducted. Disease phenotypes were defined according to the Lublin classification (18). Eligible studies were required to report comparative data based on groups stratified by baseline serum or plasma sNfL levels, where high and low groups were differentiated using explicitly defined, study-specific thresholds. Studies must have reported at least one clinical or radiological prognostic outcome after a minimum follow-up of 12 months, including confirmed disability progression, relapse-related outcomes, or new MRI activity. We excluded cross-sectional studies, studies without a comparative group, studies measuring sNfL solely in CSF, studies with a follow-up duration of less than 12 months, and studies from which effect estimates could not be extracted or calculated. The detailed search process is shown in **Figure 1**.

### 2.3 Data extraction

Two authors independently performed data extraction. In case of disagreement, a third researcher was involved in data extraction and discussion until a consensus was reached. The parameters for each study were as follows: Study identifier (first author’s name), country/region, new T2 lesions (NTL), disease worsening, ≥1 gadolinium-enhancing (Gd+) lesion, relapse within 1 year (R1Y), the cut-off value of sNfL, study category, number of participants and MS subtype.

### 2.4 Primary and secondary outcomes

NCR and ≥1 Gd+ lesion were set as primary outcomes. NCR was defined as the onset of new or acute worsening of neurological symptoms typical of MS during follow-up. ≥1 Gd+ lesion refers to the detection of one or more Gd+ lesions on a follow-up MRI scan of the brain and/or spinal cord.

R1Y, NTL and disease worsening were defined as secondary outcomes. R1Y was defined as a clinically confirmed relapse occurring within the consecutive 12-month follow-up period after the collection of the baseline serum sample used for sNfL. NTL was deemed as ​newly appearing focal areas of hyperintensity on T2-weighted or fluid-attenuated inversion recovery (FLAIR) MRI sequences that are present on a follow-up scan but were not visible on a previous baseline scan. Disease worsening was defined as a confirmed increase in the Expanded Disability Status Scale (EDSS) score or confirmed disability progression over a specified period during follow-up.

### 2.5 Assessment of risk of bias

Two authors independently appraised the quality of the included cohort studies using the Newcastle-Ottawa Scale (NOS). This tool evaluates studies across three domains: the selection of study groups, the comparability of groups, and the ascertainment of either the exposure or outcome (**Supplementary Table 1**). Any discrepancies in scoring were resolved through discussion, with a third researcher consulted to reach a consensus if necessary.

### 2.6 Risk of publication bias & sensitivity analysis

Publication bias was assessed using Egger’s regression test and Begg’s rank correlation test. Sensitivity analysis, conducted via the leave-one-out method, was performed to evaluate the robustness of the pooled results by iteratively excluding individual studies.

### 2.7 Statistical analysis

This meta-analysis was performed using STATA software, version 14.0. For all dichotomous outcomes, pooled effect estimates are reported as risk ratio (RR) with corresponding 95% confidence intervals (CI). A random-effects model, using the inverse-variance method, was employed for all primary meta-analyses to provide a more conservative estimate and account for potential heterogeneity across the included studies. Between-study heterogeneity was statistically quantified using the Cochran’s Q test (with significance set at P<0.10) and the I² statistic. An I² value greater than 50% was considered to indicate substantial heterogeneity. A two-sided p-value of less than 0.05 was considered statistically significant for all summary effect estimates.

## 3 Results

### 3.1 Selection & characteristics of included studies

According to the predetermined search strategy, 3,281 records were initially identified from online databases for the period spanning January 2015 to January 2026. Following the removal of 394 duplicate entries, 2,887 unique records underwent title and abstract screening. At this stage, 1,304 records were excluded as they pertained to animal studies, resulting in 1,583 potentially relevant articles for full-text review. Full-text articles were retrievable for 1,549 of these studies. After a detailed assessment, 1,536 studies were excluded for not meeting the predefined eligibility criteria, yielding 13 studies incorporating 12,513 patients for final inclusion in the meta-analysis (**Figure 1**) (19–31). The basic characteristics of the included studies are summarized in **Supplementary Table 2**.

### 3.2 Primary outcomes

#### 3.2.1 NCR

**Figure 2:**
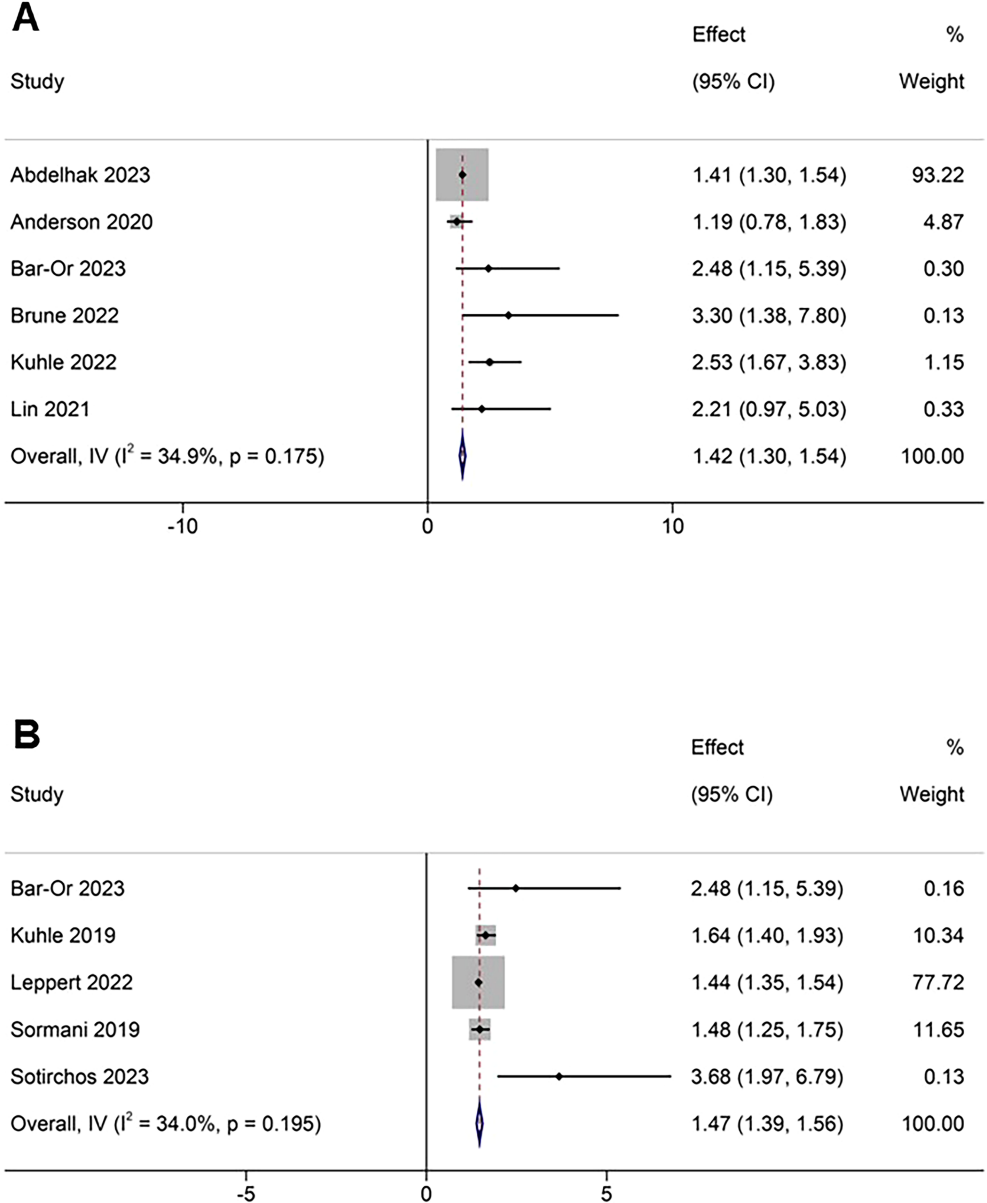
Forest plots for association between elevated sNfL and **(A)** NCR, **(B)** ≥1 Gd+ lesion.

Six studies provided evaluable data on NCR. A random-effects meta-analysis demonstrated a significantly elevated risk of NCR among patients with high sNfL concentrations compared to those with low sNfL (RR 1.42, 95% CI 1.30–1.54; P<0.01). These findings support the potential role of sNfL as a predictive biomarker for clinical relapse activity in MS. There was no substantial heterogeneity across the included studies (I^2^ = 34.9%; P = 0.175) (**Figure 2A**).

#### 3.2.2 ≥1 Gd+ lesion

Data from five studies assessing ≥1 Gd+ lesion were included in the meta-analysis. A random-effects model revealed a significantly increased risk of developing ≥1 Gd+ lesion among patients with elevated sNfL concentrations compared to those with lower levels (RR 1.47, 95% CI 1.39–1.56; P<0.01). No substantial heterogeneity was observed across studies (I²=34.0%; P = 0.195) (**Figure 2B**).

### 3.3 Secondary outcomes

#### 3.3.1 R1Y

**Figure 3:**
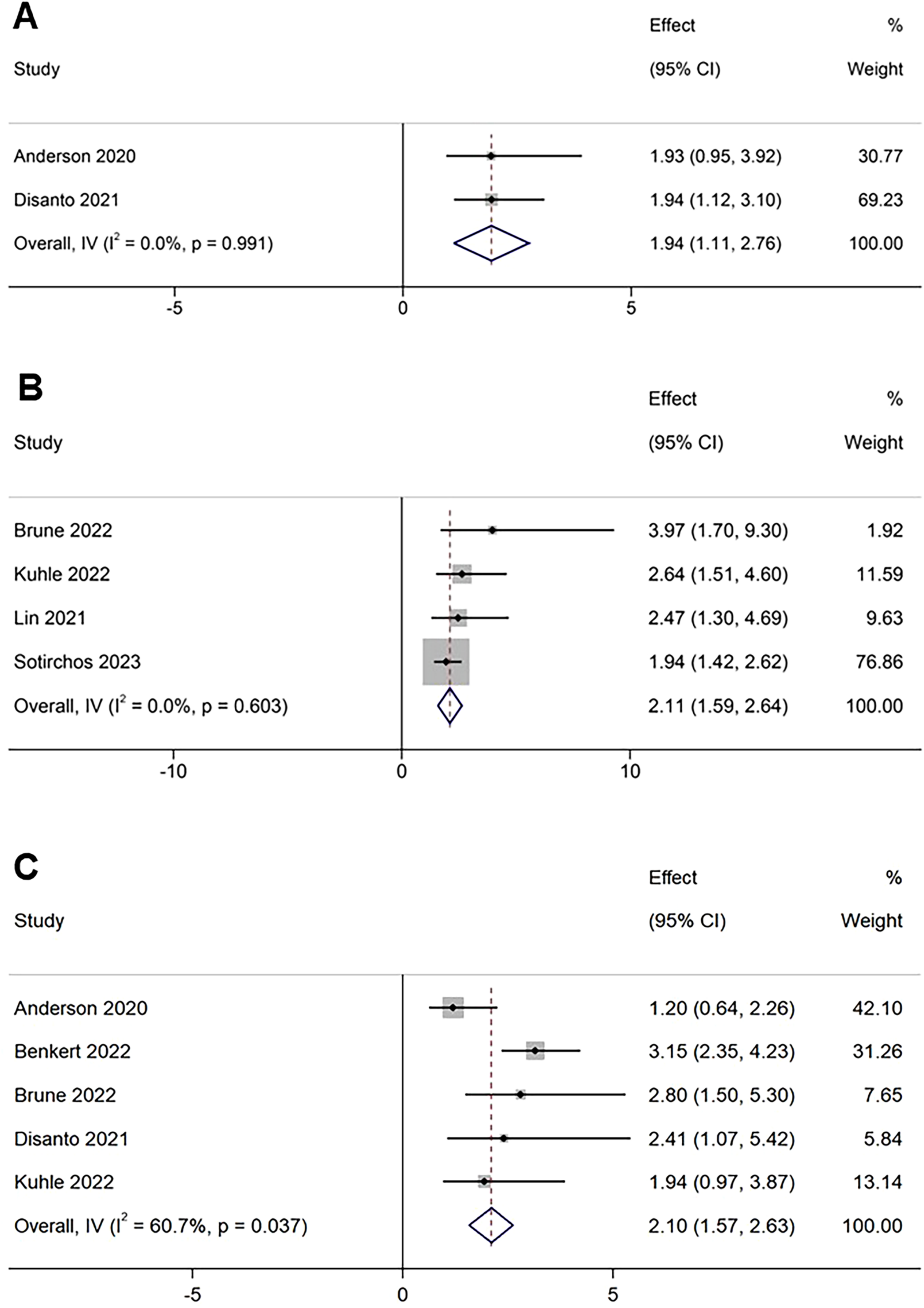
Forest plots for association between elevated sNfL and **(A)** R1Y, **(B)** NTL, **(C)** disease worsening.

Data from a limited number of studies (n=2) evaluating R1Y risk were included in the meta-analysis. A random-effects model demonstrated a significantly higher risk of R1Y among patients with elevated sNfL concentrations compared to those with lower levels (RR 1.94, 95% CI 1.11–2.76; P<0.01). No significant heterogeneity was detected across the studies (I²=0%; P = 0.991) (**Figure 3A**).

#### 3.3.2 NTL

Data from four studies assessing NTL were included in the meta-analysis. A random-effects model revealed a significantly increased risk of NTL among patients with elevated sNfL concentrations compared to those with lower levels (RR 2.11, 95% CI 1.59–2.64; P<0.01). No substantial heterogeneity was observed across studies (I²=0%; P = 0.603) (**Figure 3B**).

#### 3.3.3 Disease worsening

Data from five studies evaluating disease worsening were included in the meta-analysis. A random-effects model demonstrated a significantly increased risk of disease worsening among patients with elevated sNfL concentrations compared to those with lower levels (RR 2.10, 95% CI 1.57-2.63; P<0.01). Significant heterogeneity was observed across the included studies (I² = 60.7%; P = 0.037) (**Figure 3C**).

### 3.4 Publication bias & sensitivity analysis

Results from Egger’s and Begg’s tests indicated no substantial publication bias across the four endpoints: NCR (Egger’s test, P = 0.101; Begg’s tests, P = 0.236), ≥1 Gd+ lesion (Egger’s test, P = 0.082; Begg’s tests, P = 0.157), NTL (Egger’s test, P = 0.368; Begg’s tests, P = 0.491), and disease worsening (Egger’s test, P = 0.218; Begg’s tests, P = 0.315). However, publication bias assessment was not feasible for R1Y, owing to the limited number of included studies (n=2), which falls below the threshold for reliable statistical testing (**Supplementary Figure 1**).

Sensitivity analysis using the leave-one-out method for the four outcome indicators demonstrated that the statistical significance of the results remained unchanged after successively excluding each included study (**Supplementary Figure 2**).

## 4 Discussion

This systematic review and meta-analysis of 13 studies provides robust evidence that elevated sNfL concentrations are consistently associated with worse clinical and radiological outcomes in people with MS. The significant associations observed across all five pre-specified outcomes, ranging from short-term relapse activity to long-term disability progression, substantiate the prognostic value of sNfL as a biomarker of disease activity and progression.

Our findings align with and extend previous research in this field (32, 33). The association between sNfL and clinical relapse is consistent with smaller cohort studies that reported similar relationships, but our meta-analysis provides a more precise estimate through pooled data from multiple populations (34, 35). The strong association with Gd+ lesions reinforces the concept that sNfL elevations correlate with blood-brain barrier disruption and acute inflammatory activity. Notably, the effect size for disease worsening was higher than that for relapse activity, potentially indicating that sNfL captures not only inflammatory activity but also neurodegenerative processes that drive disability accumulation which was a distinction that has therapeutic implications given the differential responses of inflammation and neurodegeneration to current disease-modifying therapies.

The low to moderate heterogeneity observed for most outcomes enhances confidence in the robustness of our findings. However, the substantial heterogeneity for disease worsening warrants consideration. This heterogeneity may reflect clinical diversity in how disability progression was defined across studies, variations in follow-up duration, or differences in baseline patient characteristics. The consistency of associations across sensitivity analyses suggests that this heterogeneity does not undermine the primary conclusion but highlights the need for standardized outcome definitions in future studies.

The consistent prognostic performance of sNfL across multiple endpoints supports its potential integration into clinical practice and trial design (36, 37). In clinical settings, sNfL measurement could help identify patients at higher risk of disease activity who might benefit from more intensive monitoring or optimized treatment strategies, including earlier treatment escalation or initiation of early intensive therapy (38). For clinical trials, sNfL could serve as a stratification factor to ensure balanced allocation of high-risk patients or as a secondary endpoint to provide complementary evidence of treatment efficacy on neuroaxonal injury (39). The minimal publication bias detected for most outcomes strengthens the validity of these potential applications.

Importantly, the biological interpretation of sNfL should be considered in the context of the close interplay between neuroinflammation and neurodegeneration in MS. Traditionally, neuroinflammation has been viewed as the primary driver of early disease activity, while neurodegeneration was considered a later consequence (40). However, accumulating evidence suggests that neurodegenerative processes may begin concurrently with inflammatory activity, even at early stages of the disease (41, 42). Inflammatory demyelination, oxidative stress, and mitochondrial dysfunction can all contribute to early axonal injury, leading to the release of neurofilament light chain into the extracellular space (24, 43–45).

In this framework, sNfL represents a composite biomarker reflecting both acute inflammatory damage and chronic neurodegenerative processes. This dual biological relevance may explain why elevated sNfL levels are associated not only with relapse-related outcomes and MRI activity, but also with long-term disability progression (46). Therefore, sNfL may provide a unique link between inflammatory activity and irreversible neuroaxonal loss, supporting its role as a prognostic indicator across different stages of MS.

Several limitations should be acknowledged. First, the number of studies available for some outcomes (particularly R1Y, with only two studies) limited more detailed subgroup analyses or assessment of publication bias. Given the limited number of included studies, the findings for R1Y should be considered exploratory and interpreted with caution. Second, the use of study-specific thresholds for defining high versus low levels introduces methodological heterogeneity, though this approach reflects real-world variability in assay platforms and clinical contexts. Third, we could not explore potential effect modifiers such as disease phenotype, age, or treatment status in depth due to inconsistent reporting across studies. Finally, the limited number of studies precluded meaningful subgroup analyses to explore potential sources of heterogeneity.

## 5 Conclusion

Elevated sNfL levels are associated with an increased risk of clinical and radiological disease activity in MS. As a marker of neuroaxonal damage, sNfL may aid in risk stratification and treatment decision-making. While evidence for some outcomes remains limited, these findings support its role as a biomarker of disease activity. Further studies are needed to validate its clinical utility.

## Data Availability

The original contributions presented in the study are included in the article/**Supplementary Material**. Further inquiries can be directed to the corresponding author.

## Supplementary material

Supplementary Figure 1 Publication bias assessment for **(A)** NCR, **(B)** ≥1 Gd+ lesion, **(C)** NTL, **(D)** disease worsening.

Supplementary Figure 2 Leave-One-Out sensitivity analysis for **(A)** NCR, **(B)** ≥1 Gd+ lesion, **(C)** NTL, **(D)** disease worsening.
