# Supplementary figures and images for: Serum neurofilament light chain and multiple sclerosis prognosis: a systematic review and meta-analysis
Source: Front Immunol. 2026 Apr 28;17:1818869. doi: 10.3389/fimmu.2026.1818869 (PMC13161020; doi:10.3389/fimmu.2026.1818869)

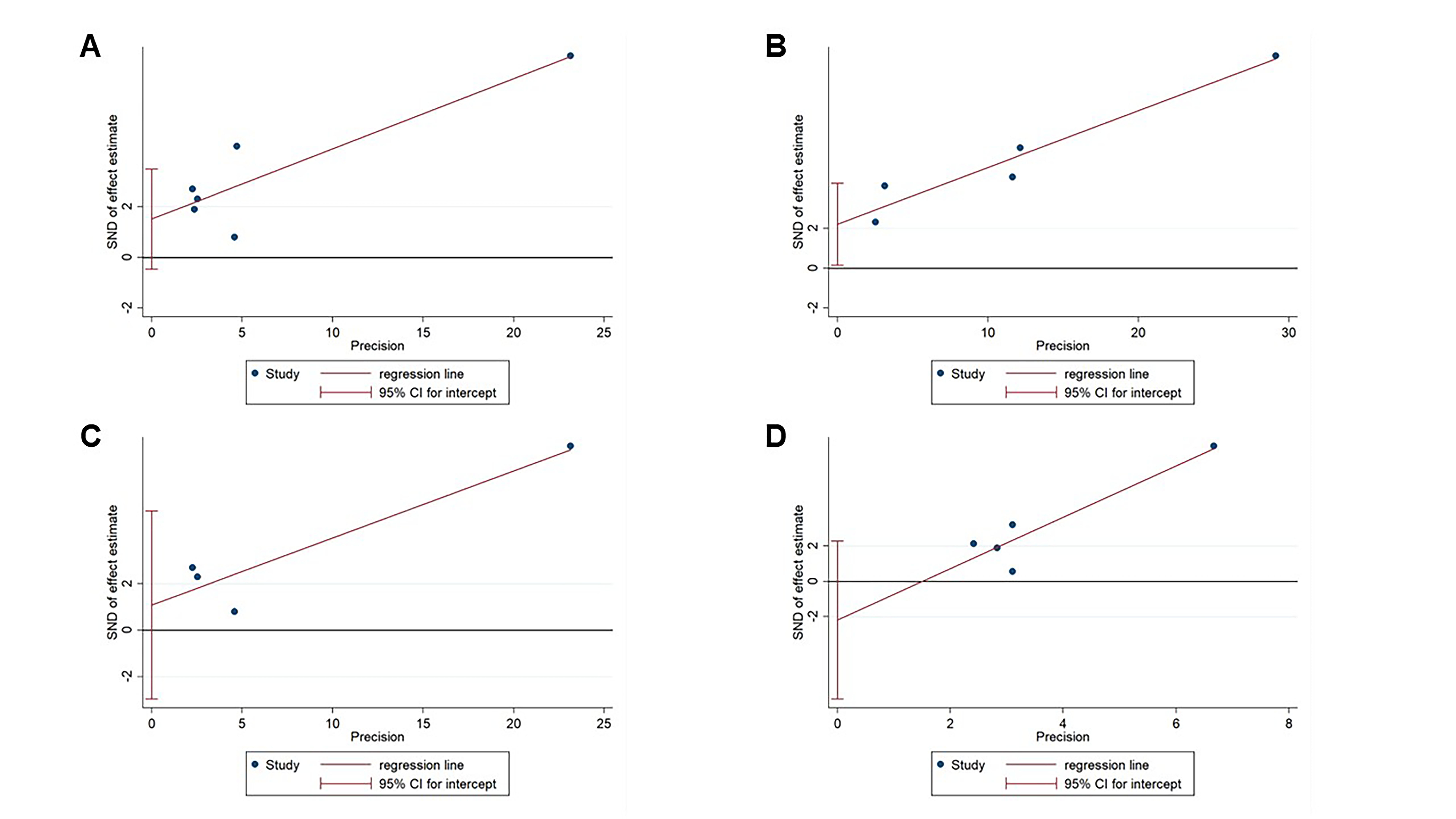

Supplement: Supplementary Figure 1 — Publication bias assessment for (A) NCR, (B) ≥1 Gd+ lesion, (C) NTL, (D) disease worsening. [file Image1.jpeg]

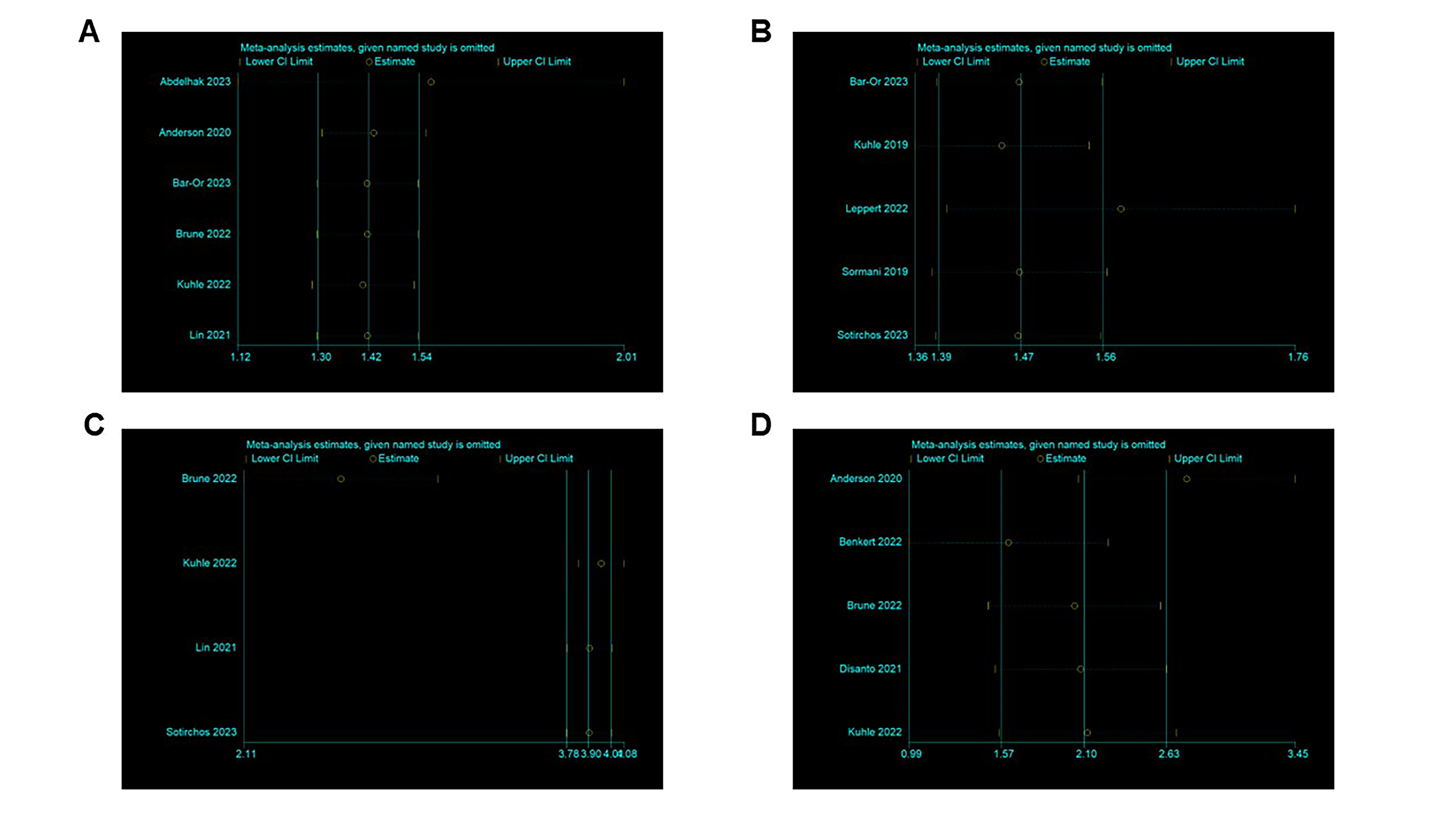

Supplement: Supplementary Figure 2 — Leave-One-Out sensitivity analysis for (A) NCR, (B) ≥1 Gd+ lesion, (C) NTL, (D) disease worsening. [file Image2.jpeg]
