# Supplementary material for: Serum neurofilament light chain and multiple sclerosis prognosis: a systematic review and meta-analysis
Source: Front Immunol. 2026 Apr 28;17:1818869. doi: 10.3389/fimmu.2026.1818869 (PMC13161020; doi:10.3389/fimmu.2026.1818869)
Supplement: Supplementary file 3 [file Table1.docx]

**Supplementary Table 1.** Newcastle-Ottawa Scale scores for the 13 included cohort studies

| Study | Representativeness of the exposed cohort | Selection of the non-exposed cohort | Ascertainment of exposure | Demonstration that outcome of interest was not present at start of study | Comparability of cohorts on the basis of the design or analysis | Assessment of outcome | Was follow-up long enough for outcomes to occur | Adequacy | Total score |
| --- | --- | --- | --- | --- | --- | --- | --- | --- | --- |
| Abdelhak et al., 2023 | ★ | ★ | ★ | ★ | ★ | ★ | ★ | ★ | 8 |
| Anderson et al., 2020 | ★ | ★ | ★ | ★ | ★ | ★ | ★ | ★ | 8 |
| Bar-Or et al., 2023 | ★ | ★ | ★ | ★ | ★ | ★ | ★ | ★ | 8 |
| Benkert et al., 2022 | ★ | ★ | ★ | ★ | ★★ | ★ | ★ | ★ | 9 |
| Brune et al., 2022 | ★ | ★ | ★ | ★ | ★★ | ★ | ★ | ★ | 9 |
| Cutter et al., 2023 | ★ | ★ | ★ | ★ | ★ | ★ | ★ | ★ | 8 |
| Disanto et al., 2021 | ★ | ★ | ★ | ★ | ★ | ★ | ★ | ★ | 8 |
| Kuhle et al., (1), 2019 | ★ | ★ | ★ | ★ | ★ | ★ | ★ | ★ | 8 |
| Kuhle et al., (2), 2022 | ★ |  | ★ | ★ | ★ | ★ | ★ |  | 6 |
| Leppert et al., 2022 | ★ | ★ | ★ | ★ | ★ | ★ | ★ | ★ | 8 |
| Lin et al., 2021 |  | ★ | ★ | ★ | ★ | ★ | ★ | ★ | 7 |
| Sormani et al., 2019 | ★ | ★ | ★ | ★ | ★ | ★ | ★ | ★ | 8 |
| Sotirchos et al., 2023 | ★ | ★ | ★ | ★ | ★ | ★ | ★ | ★ | 8 |
