# Supplementary material for: Serum neurofilament light chain and multiple sclerosis prognosis: a systematic review and meta-analysis
Source: Front Immunol. 2026 Apr 28;17:1818869. doi: 10.3389/fimmu.2026.1818869 (PMC13161020; doi:10.3389/fimmu.2026.1818869)
Supplement: Supplementary file 4 [file Table2.docx]

**Supplementary Table 2.** Included Studies Baseline Characteristics.

| **Study** | **Country/Region** | **Number of participants** | **MS subtype** | **Absolute cut-off** | **Definition of cut-off** | **Criteria of disease worsening** | **Study category** |
| --- | --- | --- | --- | --- | --- | --- | --- |
| Abdelhak et al., 2023 | Europe and North America | 1899 | All multiple sclerosis subtypes | Not reported | Not reported | - | Prospective cohort study |
| Anderson et al., 2020 | UK | 164 | Relapsing multiple sclerosis | 13.7 pg/ml | Median | EDSS | Prospective cohort study |
| Bar-Or et al., 2023 | Austria, Bulgaria, Czech Republic, Estonia, Latvia, Lithuania, Russian, Spain, and the USA | 284 | Relapsing multiple sclerosis | 9.1 pg/ml | Median | - | Prospective cohort study |
| Benkert et al., 2022 | Europe and North America | 5390 | All multiple sclerosis subtypes | Not reported | First quartile | EDSS | Prospective cohort study |
| Brune et al., 2022 | Norway | 226 | All multiple sclerosis subtypes | 8 pg/ml | 75^th^ percentile | EDSS | Prospective cohort study |
| Cutter et al., 2023 | USA | 675 | Relapsing multiple sclerosis | 16 pg/ml | Median | - | Prospective cohort study |
| Disanto et al., 2021 | Switzerland | 254 | All multiple sclerosis subtypes | Not reported | Linear generalized estimating equation models | EDSS | Prospective cohort study |
| Kuhle et al., (1), 2019 | 16 countries | 162 | Relapsing multiple sclerosis | Not reported | Not reported | - | Prospective cohort study |
| Kuhle et al., (2), 2022 | Nor reported | 589 | Relapsing multiple sclerosis | 60 pg/ml | Geometric mean | EDSS | Prospective cohort study |
| Leppert et al., 2022 | 31 countries | 1452 | All multiple sclerosis subtypes | 30 pg/ml | Geometric mean | - | Prospective cohort study |
| Lin et al., 2021 | Germany | 78 | All multiple sclerosis subtypes | Not reported | 80^th^ percentile | - | Prospective cohort study |
| Sormani et al., 2019 | 22 countries | 102 | Relapsing multiple sclerosis | 30 pg/ml | Geometric mean | - | Prospective cohort study |
| Sotirchos et al., 2023 | USA, Europe | 1238 | All multiple sclerosis subtypes | Not reported | Not reported | - | Prospective cohort study |
